# Prognostic assessment of liver cirrhosis and its complications: current concepts and future perspectives

**DOI:** 10.3389/fmed.2023.1268102

**Published:** 2023-09-14

**Authors:** Dilan Elcin Bozal, Tobias Goeser, Philipp Kasper

**Affiliations:** Department of Gastroenterology and Hepatology, Faculty of Medicine and University Hospital Cologne, University of Cologne, Cologne, Germany

**Keywords:** cirrhosis, prognosis, scoring systems, ACLF, AD, portal hypertension, LSM, SSM

## Abstract

Liver cirrhosis is an irreversible stage of chronic liver disease with varying clinical course. Acute decompensation of liver cirrhosis represents a watershed in prognosis and is characterized by the occurrence of clinical complications such as ascites, jaundice, hepatic encephalopathy, infections, or portal-hypertensive hemorrhages. Emergent data indicate that an acute decompensation can be subdivided into stable decompensated cirrhosis (SDC), unstable decompensated cirrhosis (UDC), pre-acute-on chronic liver failure (pre-ACLF) and acute-on chronic liver failure (ACLF), while the mortality risk varies greatly between the respective subgroups. ACLF is the most severe form of acutely decompensated cirrhosis and characterized by the development of organ failure(s) and a high short-term mortality. Due to the dynamic disease course of acute decompensation, it is paramount to detect patients at particular risk for severe complications those at high risk for developing ACLF as early as possible in order to initiate optimal management. This review describes new concepts and perspectives in the definition and classification of decompensated cirrhosis and provides on overview on emerging predictive scoring systems, non-invasive measurement methods and new biomarkers, which allow an early identification of patients with acute decompensation at risk.

## Introduction

Liver cirrhosis is the final stage of chronic liver disease and characterized by an irreversible replacement of liver parenchyma with fibrotic tissue and regenerative nodules (1). The major causes of cirrhosis include hepatitis B virus (HBV) and hepatitis C virus (HCV) infection, alcohol-associated liver disease (ALD), and non-alcoholic fatty liver disease (NAFLD). Though viral hepatitis remains globally the leading cause of cirrhosis, the prevalence and incidence of alcoholic cirrhosis and NAFLD-related cirrhosis are rising in several regions of the world during the past decades due to an increased alcohol consumption and the ongoing epidemic of obesity (2, 3).

## Clinical course of cirrhosis: from compensated cirrhosis to acute-on-chronic liver failure

The course of liver cirrhosis can be divided into a compensated and a decompensated stage (4). Compensated cirrhosis (CC) is considered as a stable state of disease where clinical symptoms are often absent or minor. The 1-year mortality rate of CC is estimated to be <5% (5).

The manifestation of clinical complications such as ascites, jaundice, hepatic encephalopathy, portal hypertensive bleeding (e.g., variceal hemorrhage), infections or any combination of these disorders define an acute decompensation (AD) (1, 6). An acute decompensating event represents a watershed in prognosis. Ascites occurs as the most common first decompensating event, with a drastic increase in 1-year mortality rising up to 20%, while 1-year mortality of acute decompensation due to infections even rises to over 50% (4, 7–9). Precipitating events for decompensation include bacterial or viral infections, aggravations of underlying liver disease (e.g., hepatitis B flare), alcoholic hepatitis, and drug-induced liver injury (4, 6, 9).

While the classification into a compensated and decompensated form has been established in everyday clinical practice for decades, recent data have shown that this dichotomous type of classification is an oversimplification (10). Since decompensated cirrhosis encompasses several different prognostic subgroups of patients with varying risk profiles, a more specific classification is needed.

Two landmark studies that have significantly improved the understanding of acute decompensation in recent years, have been the Consortium on Acute-on-Chronic Liver Failure in Cirrhosis (CANONIC) and the Predicting-of-Acute-on-Chronic Liver Failure in Cirrhosis (PREDICT) studies.

The results of the CANONIC and the PREDICT studies provided detailed insights on disease progression and its course and allow us today to classify different stages of decompensated liver cirrhosis more precisely.

The aim of the CANONIC study was to identify a definition and diagnostic criteria for the most severe form of acute decompensation, termed acute-on-chronic liver failure (ACLF). ACLF represents a distinct form of acutely decompensated liver cirrhosis, which is characterized by the onset of (extra-hepatic) organ failure(s) and a high short-term mortality (11–13). The CANONIC study found that ACLF is a dynamic syndrome, which can improve or conversely worsen and identified systemic inflammation as a major driver of ACLF.

The PREDICT study added further information on the course of AD and provides the basis for establishing a new terminology by uncovering three different clinical courses of acutely decompensated cirrhosis in patients without ACLF. According to the PREDICT study, AD can be categorized into stable decompensated cirrhosis (SDC), unstable decompensated cirrhosis (UDC), and a pre-ACLF stage.

Patients with SDC are characterized by the occurrence of typical complications such as ascites, low systemic inflammation, and facility of rapid recompensation. Patients with SDC typically do not require re-hospitalization due to further decompensation events, and serious organ failure is rarely observed in SDC. The 3-month mortality of SDC is about 0% and the 1-year mortality rate in SDC about 10% (9, 14).

UDC is associated with significant portal hypertension, gastrointestinal bleeding episodes and increased incidence of bacterial infections, resulting in further decompensation events. After the initial decompensation event, UDC is defined by the need for at least one further hospital readmission. Although organ dysfunction occurs more frequently in UDC than in SDC (29% brain dysfunction, 19% circulatory failure, and 16% liver dysfunction), patients with UDC do not usually develop ACLF. The 90-day mortality of UDC is about 21% and the 1-year mortality in UDC is about 36% (9, 14).

Patients in the pre-ACLF stage typically develop ACLF during follow-up and have a 3-month mortality of about 53% and a significantly higher 1-year mortality of more than 65%. While the UDC stage is characterized by increased portal hypertension and frequent occurrence of gastrointestinal bleeding, the pre-ACLF stage is characterized by significantly higher systemic inflammation and can thereby distinguished from SDC and UDC. Inflammatory biomarkers include leukocyte count, C-reactive protein (CRP), and interleukin-6 (IL-6), all of which showing a successive increase with progression of decompensation severity (12, 15).

**Figure 1:**
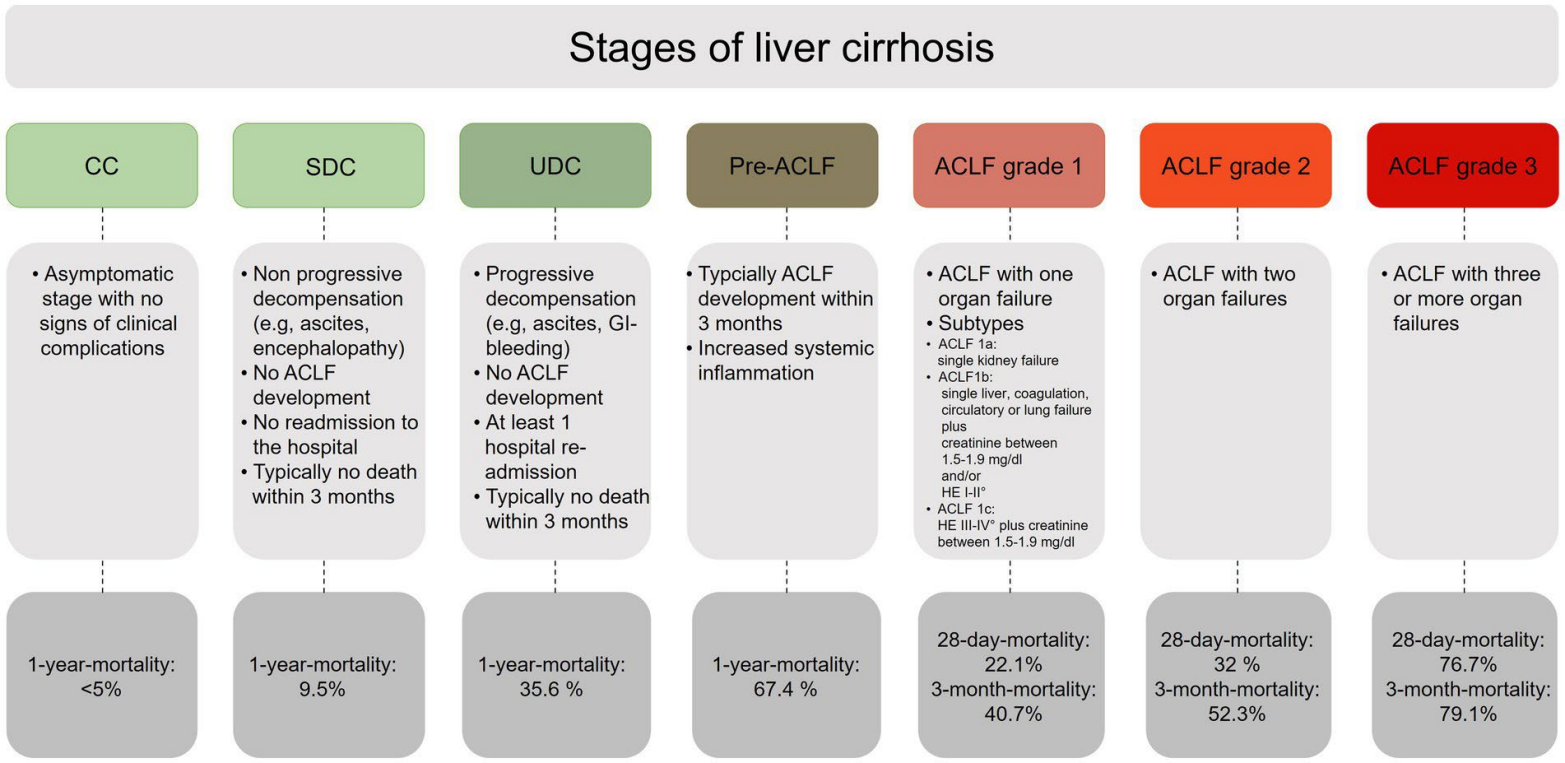
Overview of the different stages of liver cirrhosis. CC, compensated cirrhosis; SDC, stable decompensated cirrhosis; UDC, unstable decompensated cirrhosis; pre-ACLF, pre-acute-on-chronic liver failure; ACLF, acute-on-chronic liver failure.

Thus, a modern classification of cirrhosis should include the following disease stages: CC, SDC, UDC, Pre-ACLF, and ACLF (Figure 1). However, it must be kept in mind that these stages do not have to follow each other in an obligatory sequential manner, since the disease course of cirrhosis can vary tremendously.

Recently, the European Association for the Study of Liver Disease (EASL) published their updated ACLF clinical practice guidelines, whose recommendations for the classification of AD were largely based on the findings from the CANONIC and PREDICT studies (16). According to the recent EASL-guidelines, ACLF is characterized by functional organ failure of the six major organ systems (liver, kidney, brain, coagulation, circulation, and respiration) and systemic inflammation, induced by acute hepatic or extrahepatic precipitating factors, or both. Within the updated guidelines, AD is also divided into the subtypes, as described above (16).

## Prognostic assessment of decompensated liver cirrhosis

In addition to establish a diagnosis, prognostic evaluation is an essential part of any disease. Besides its role in informing patients, prognostic evaluation also forms the basis for any decision-making process by clinicians.

In the field of liver cirrhosis, several different classification models exist. In clinical practice, the Child-Turcotte-Pugh (CTP) classification has become widely accepted for risk assessment in cirrhotic patients over the past decades (8, 15). Even the original purpose of this scoring system was the risk assessment in patients undergoing surgical porto-systemic shunt operation, it was later also used for stratifying patients for liver transplantation (17, 18). However, due to several limitations like the subjective assessment of different score components (e.g., ascites) the CTP score was subsequently superseded by the more reproducible Model for End-Stage Liver Disease (MELD) score (19).

The MELD score, which was initially developed for short-time mortality analysis in patients undergoing transjugular-intrahepatic-portosystemic-shunt (TIPS) implantation, is based on laboratory parameters (serum creatinine, bilirubin, INR) and ranges from 6 to 40. The MELD score can be used to predict 3-month mortality in patients with end-stage liver disease, even more accurately than the CTP score (8, 19, 20). Since sodium (Na) levels represent another independent predictor of mortality in cirrhosis, this marker can be incorporated into the ordinary MELD score and the MELD-Na score can be calculated. The MELD-Na score is currently used to determine organ allocation priorities for liver transplantation in the United States, whereas the ordinary MELD score is used in the European Eurotransplant (ET) region to allocate liver grafts (19).

However, the MELD score and MELD-Na score have some limitations that are currently the subject of debate. Studies have shown that renal function is not adequately reflected by serum creatinine and that individuals with lower muscle mass (e.g., cirrhotic patients with sarcopenia) as well as women who have less muscle mass compared with male counterparts are disadvantaged by using the MELD score for transplant allocation and prioritization. Furthermore, the MELD-Na score does not accurately predict risk in patients with ACLF (19).

Due to existing limitations new models of the MELD score, including but not limited to MELD-lactate, MELD-Na with transient elastography (TE), and the MELD 3.0 score including female sex and serum albumin as additional variables, has been developed in recent years to improve mortality prediction and allocation prioritization in liver transplantation (21).

## Prognostic scoring systems in AD and ACLF

In the context of a dynamic acute decompensation and ACLF, new scoring systems have also been developed in recent years to identify patients at particular risk as early as possible. This scoring systems include the CLIF-C AD and CLIF-ACLF score.

**Figure 2:**
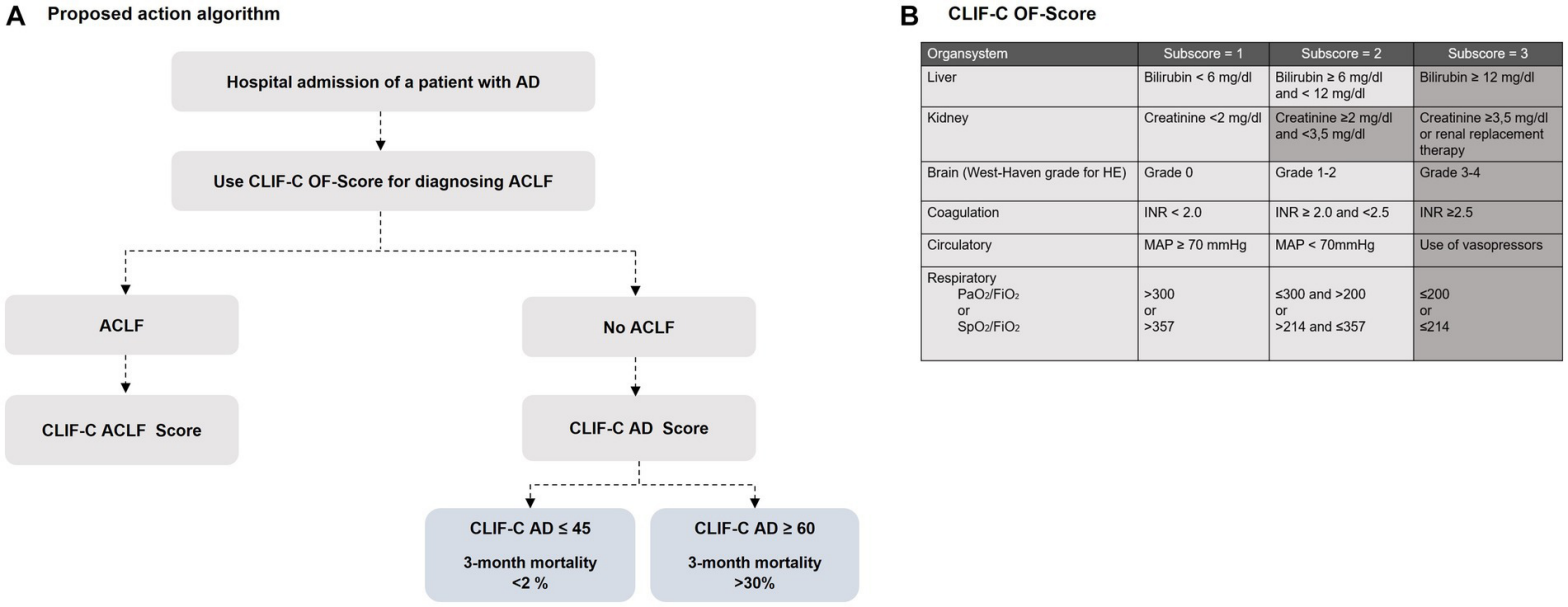
**(A)** A proposed algorithm for using the EASL CLIF-Consortium predictive score scoring systems for identifying cirrhotic patients with AD and ACLF [modified from Jalan et al. (22)]. The following formulas are used for calculation: CLIF-C Acute-on-chronic liver failure (CLIF-C ACLF) formula: CLIF ACLF score = 10 × (0.33 × CLIF-OF score + 0.44 × Age [years] + 0.63 × ln(WBC [109/L] − 2) (23); CLIF-C Acute decompensation (CLIF-C AD) formula: CLIF-C AD score = (10 × 0.03 × Age [years]) + (0.66 × ln(SCr [mg/dL]) + 1.71 × ln(INR) + 0.88 × ln(WBC [109/L]) − 0.05 × Na [mmol/L] + 8 (22). **(B)** Overview of the Chronic Liver Failure-Consortium Organ Failure scale ()-score with definition criteria for organ failure. Shadowed areas define criteria for the diagnosis of organ failure. HE, Hepatic encephalopathy; FiO2, fraction of inspired oxygen; PaO2, partial pressure of arterial oxygen; SpO2, pulse oximetric saturation.

Every patient who is admitted to hospital with acutely decompensated liver cirrhosis should evaluated immediately for the presence of (un-)complicated acute decompensation or ACLF (Figure 2A). The diagnosis of ACLF can be established if hepatic or extrahepatic organ failure is present, which should be defined according to the CLIF-C Organ Failure (CLIF-C-OF) score (24). The CLIF-C OF scoring system uses different clinical and biochemical criteria to define failure of the liver, kidneys, brain, coagulation, circulation, and/or respiration (Figure 2B).

If the diagnosis ACLF is established, the grade of an ACLF depends on the number of underlying organ failures. ACLF grade 1a is present in isolated renal failure with a serum creatinine level ≥ 2 mg/dL, while ACLF grade 1b is present in case of isolated liver, coagulation, circulatory, or respiratory failure with a serum creatinine level of 1.5–1.9 mg/dL and/or grade I-II encephalopathy. ACLF grade 1c, in turn, is present in isolated cerebral failure with hepatic encephalopathy grade III-IV and a serum creatinine level of 1.5–1.9 mg/dL. If there are two or three organ failures, a grade 2 or grade 3 ACLF can be diagnosed depending on the number of concomitant organ failures (13, 16).

To predict the mortality risk in patients with ACLF, it is recommended to apply the CLIF-C ACLF score, as this specific score reached a significantly higher predictive accuracy for mortality than the previously applied standard scoring systems, such as MELD, MELD-Na and the CTP score. After specific treatment measurements and multiorgan support have been initiated in patients with ACLF, a sequential use of the CLIF-C ACLF score during the hospital stay is recommended to evaluate treatment response, but also to identify patients in whom intensive care is likely to be futile despite maximal treatment efforts (25). Patients with a CLIF-C ACLF score ≥ 70 had a 100% mortality within 28-days after hospitalization (26), so that in particulary these cases, interdisciplinary evaluation is required to determine the extent to which intensive medical care should be continued or palliative regimens should be initiated (26).

If the diagnostic criteria of an ACLF are not met, an AD can be diagnosed, and the CLF-C AD score should be calculated for severity assessment.

The CLIF-C-AD score was developed to predict the 3-month mortality in patients with acute decompensated liver cirrhosis (23) and is recommended to identify vulnerable patients at high mortality risk. With a potential scoring range of 0–100, patients with a CLIF-C AD ≤45 had a 3-month mortality less than 2%, whereas a score value ≥60 indicated a higher 3-month mortality of 30%. Since the CLIF-C-AD show superiority in predicting mortality to previously mentioned scores (MELD, CTP) it should be routinely calculated in all patients with AD (23).

In contrast to the previously available scores, the CLIF-C AD and CLIF-C ACLF scores also include age and leukocyte count, so that for the first-time systemic inflammation, which represents an important driver of disease progression in decompensation, is also considered as a prognostic parameter.

## Emerging scoring systems and biomarkers

In addition to define and classifying the severity of AD and ACLF, scoring systems and biomarkers that predict the (future) risk of AD are also of great interest in everyday clinical practice, since early detection of transition to a decompensated stage of cirrhosis would enable timely targeted therapeutic interventions.

Recently Schneider and colleagues published the Early-Prediction-of-Decompensation (EPOD) score to predict the probability of decompensation within the next 3-years. In a large, multi-center cohort of 6,049 patients with compensated cirrhosis, the authors demonstrated that the EPOD score, which incorporates albumin level, platelet count, and bilirubin concentration, provides a good prediction tool for the risk of decompensation, and outperforms the established MELD and Child-Pugh score in predicting the risk of decompensation (27).

Other scoring systems to identify patients at risk for decompensation include the Albumin-Bilirubin- (ALBI) and the Firbosis-4 (FIB)-4 score. The ALBI score was developed to assess liver function in patients with hepatocellular carcinoma (HCC) undergoing liver surgery and has also been successfully applied to the prediction of survival and hepatic decompensation in patients with non-malignant liver diseases (22). Recent data has shown that the ALBI score can detect even slight deteriorations in liver function when compared to the Child-Pugh or MELD scores, making it a promising tool for early detection of AD.

Since the extent of fibrosis is closely associated with the risk of liver-related complications, non-invasive tests for fibrosis measurement such as the FIB-4 index, are also suitable for risk assessment of hepatic decompensation. The FIB-4 index is a reliable and useful predictor of the degree of liver fibrosis in patient with NAFLD and can also be used to predict the risk of liver-related complications and adverse outcomes (28–30). The combination of both scoring systems (ALBI-FIB-4 score) also shows a high predictive power for decompensation and represents a promising tool (31).

Nonetheless, the transferability or applicability of existing prognostic scores is not always satisfactory, as individual factors remain unconsidered. Therefore, it is essential to assess the overall situation of each patient individually and to use these scores as complementary tools for predicting mortality.

Besides scoring systems, there are also serum biomarker, which can be used for predicting prognosis and hepatic decompensation. Recently, Gurbuz et al. analyzed predictive biomarkers of decompensated cirrhosis by using untargeted serum proteomics and identified significantly lower serum concentrations of albumin, transferrin, pseudocholinesterase, transthyretin, and apolipoprotein AI in patients with cirrhosis compared with healthy individuals. Here, transferrin, pseudocholinesterase, and apolipoprotein AI show a stage-dependent decrease in serum concentration. In addition, the authors demonstrated that patients with ACLF and higher serum levels of transthyretin have a better prognosis (32).

In a recent study including 444 hospitalized patients with decompensated cirrhosis, Juanola and colleagues demonstrated, that urinary fatty-acid-binding-protein (L-FABP) levels, as an indirect marker of hepatic inflammation, were independently associated with the 3-month clinical course in patients with decompensated cirrhosis, in terms of mortality and ACLF development. Therefore, urinary L-FABP seems to be another prognostic biomarker (33).

The role of serum bile acids as marker for AD and ACLF in patients with non-cholestatic cirrhosis, is also currently being investigated, since retention of bile acids and disrupted bile acid homeostasis plays a central role in hepatic damage. In a recent prospective cohort study including 143 patients with cirrhosis Horvatits et al. demonstrated, that serum bile acids were significantly associated with AD and ACLF and represents additional marker for risk stratification regarding new onset of AD and ACLF in cirrhotic patients (34).

However, many of these promising biomarkers are expensive and not available in clinical laboratory routine, and further prospective studies in Iarger cohorts are needed.

## Non-invasive assessment of portal hypertension

Since, portal hypertension is the key driver and a proven predictor of hepatic decompensation in patients with advanced chronic liver disease, its invasive and non-invasive measurement is another important method for risk stratification.

Hepatic venous pressure gradient (HVPG) measurement is the current gold-standard procedure to determine the presence of clinically significant portal hypertension (CSPH), which is defined as an HVPG ≥10 mmHg. While a HVPG ≥10 mmHg is associated with an increased risk for the development of gastro-osephageal varices, a score of >12 mmHg with an increased risk of variceal bleeding (35–37). Though the concept of CSPH is HVPG-based, non-invasive testing methods, such as the measurements of liver and spleen stiffness, are also eligible to identify CSPH and of growing interest in clinical practice.

### Liver stiffness measurement

In patients with virus- and/or alcohol-related CC and non-obese (BMI <30 kg/m^2^) NASH-related advanced chronic liver disease, a LSM value by transient elastography of ≥25 kPa is sufficient to rule in CSPH, defining the group of patients at risk for endoscopic signs of portal hypertension (e.g., gastro-esophageal varices) and at higher risk of decompensation (35, 37). While a single baseline measurement is sufficient to identify patients at risk, repetitive measurements of LSM should be performed to predict the risk of future hepatic decompensation more precisely.

Recently, Semmler et al. demonstrated, that longitudinal dynamic changes in repeated LSM enables a more accurate risk prediction for decompensation and liver-related death in a retrospective cohort study including 2,508 patients than a single measurement (38). Specifically, a 20% increase/decrease of LSM in patients with advances liver disease indicates a ~ 50% increased/decreased risk of hepatic decompensation and liver-related death (38).

### Spleen stiffness measurement

In addition to LSM, measurement of spleen stiffness measurement (SSM) may also be applied for risk prediction of AD, as splenomegaly results from passive congestion due to portal hypertension and recent data have shown a positive correlation between HVPG and spleen stiffness (37, 39). SSM seems to be an elegant and non-invasive method for detecting CSPH and objectifying short-time dynamic changes in portal circulation, i.e., after TIPS implantation (37, 40–42). According to the Baveno VII consensus guidelines, SSM can be also used in to rule-out and rule-in CSPH (SSM <21 kPa and SSM >50 kPa, respectively). Recently, Yu et al. developed an artificial intelligence-driven spleen-based model to identifying patients with compensated cirrhosis, which are at higher risk of decompensation using a quantitative 3-dimensional (3D) volumetric analysis of the spleen. In their study, a spleen volume > 364 cm^2^ has been associated as a predictor for decompensation (43).

Unfortunately, the regional availability of TE to perform LSM and SSM is limited so far. However, if available, SSM should be performed in all cirrhotic patients in addition to LSM, and further investigation should be conducted to determine whether there are specific cut-offs that can optimally predict the risk of decompensation as well as success of re-compensation by specific treatment measurements lowering portal hypertension.

## Conclusion

The early identification of the patients with acutely decompensated cirrhosis who are at high risk of mortality and ACLF development remains an unmet clinical need. Modern scoring systems such as the CLIF-C AD and ACLF scores are valuable tools in risk assessment and should be determined as standard in all cirrhotic patients after hospitalization. Future studies are needed to investigate the extent of sequential and non-invasive measurement to predict decompensation and whether a combination of a biomarker and non-invasive measurement method is an approach to optimize risk prediction in cirrhosis.

## Author contributions

DB: Visualization, Writing – original draft. TG: Writing – review & editing, Supervision, Resources. PK: Conceptualization, Supervision, Writing – review & editing.

## Conflict of interest

The authors declare that the research was conducted in the absence of any commercial or financial relationships that could be construed as a potential conflict of interest.

## Publisher’s note

All claims expressed in this article are solely those of the authors and do not necessarily represent those of their affiliated organizations, or those of the publisher, the editors and the reviewers. Any product that may be evaluated in this article, or claim that may be made by its manufacturer, is not guaranteed or endorsed by the publisher.
